# The generalised anxiety stigma scale (GASS): psychometric properties in a community sample

**DOI:** 10.1186/1471-244X-11-184

**Published:** 2011-11-22

**Authors:** Kathleen M Griffiths, Philip J Batterham, Lisa Barney, Alison Parsons

**Affiliations:** 1 Depression & Anxiety Consumer Research Unit, Centre for Mental Health Research; The Australian National University, Acton, Canberra, ACT, Australia; 2 Centre for Mental Health Research, The Australian National University, Acton, Canberra, ACT, Australia

## Abstract

**Background:**

Although there is substantial concern about negative attitudes to mental illness, little is known about the stigma associated with Generalised Anxiety Disorder (GAD) or its measurement. The aim of this study was to develop a multi-item measure of Generalised Anxiety Disorder stigma (the GASS).

**Methods:**

Stigma items were developed from a thematic analysis of web-based text about the stigma associated with GAD. Six hundred and seventeen members of the public completed a survey comprising the resulting 20 stigma items and measures designed to evaluate construct validity. Follow-up data were collected for a subset of the participants (n = 212).

**Results:**

The factor structure comprised two components: Personal Stigma (views about Generalised Anxiety Disorder); and Perceived Stigma (views about the beliefs of most others in the community). There was evidence of good construct validity and reliability for each of the Generalised Anxiety Stigma Scale (GASS) subscales.

**Conclusions:**

The GASS is a promising brief measure of the stigma associated with Generalised Anxiety Disorder.

## Background

It is well recognised that mental disorders are associated with stigmatising attitudes [1-3]. There is some evidence that such stigma can be associated with increased psychological distress, demoralisation and isolation and reduced employment and accommodation opportunities [2-4]. It may also serve as a barrier to help seeking for mental health problems [5,6]. It has been claimed that people with anxiety disorders 'can be subject to stigmatisation in the same way as any other disorder' [7] and that such stigma serves as a barrier - at an individual level - to receiving effective treatment for anxiety [8]. However, to date most studies of stigma associated with mental disorders have focused on schizophrenia or depression. Little attention has been paid to anxiety stigma and to our knowledge there are no validated measures of the public's personal or perceived levels of stigma with respect to Generalised Anxiety Disorder (GAD). This is a significant limitation given that GAD is common, debilitating [9], and frequently untreated with research studies showing a median treatment gap for GAD of 57.5% [10].

One approach to the lack of a GAD anxiety stigma scale might be to evaluate the validity and reliability of modified versions of existing measures of the stigma associated with mental illness in general (e.g., Corrigan et al, 2004) or specific mental disorders such as depression [eg, [11,12]]. However, there is a risk that this approach might fail to reflect the elements, if any, that are unique to anxiety disorder. In addition, consideration of some scales indicates that the items have limited relevance to generalised anxiety disorder. For example, the Devaluation-Discrimination scale is strongly focused on the attributes of people with a history of psychiatric admission whereas only a very small minority of people with an anxiety disorder (2%) are hospitalised for their condition [13].

Accordingly, we report here the development and preliminary validation of a new multi-item measure of Generalised Anxiety Disorder stigma. Previously, we developed the Depression Stigma Scale (DSS) comprising two subscales, the DSS-Personal, a measure of a respondent's personal attitudes to depression, and the DSS-Perceived, a measure of a respondent's beliefs about the attitudes of others to depression [11,12]. The aim of the present study was to develop an analogue to the DSS: the Generalised Anxiety Stigma Scale (GASS).

## Methods

This study involved a quantitative survey of attitudes of members of the Australian community. It included (i) The 'Community Attitudes to Mental Health questionnaire' comprising a series of 10 personal and 10 perceived GAD stigma items together with measures designed to validate the new anxiety stigma scales; and (ii) a 4-month follow-up survey designed to assess the reliability of the new scales. The study was approved by the ANU Human Research Ethics Committee.

### Participants

In December 2009, the survey was sent by post to a total of 5000 residents aged between 18 and 65 years randomly sampled from the Australian electoral roll. Of these, 2500 participants resided in an urban region of a major city (the electorate of Banks in Western Sydney) and the remainder were randomly selected from a rural electorate (Calare in New South Wales). Registration on the electoral roll is compulsory for Australian citizens. A total of 617 citizens (12.3% excluding those which were returned unopened) completed the questionnaire. Four hundred and forty (71.3%) of these respondents indicated a willingness to complete a follow-up questionnaire. Of these, the 300 respondents who provided complete data on the first survey were sent the follow-up survey; 212 (70.6%) returned the completed follow-up questionnaire.

### Procedure and Survey

The Community Attitudes to Mental Health survey was divided into five sections. Section A comprised measures of demographic status, Section B consisted of commonly employed measures of personal and perceived stigma associated with (generic) mental illness and Section C contained measures of current levels of anxiety, depression, and psychological distress. Section D presented a labelled vignette depicting a character with a Generalised Anxiety Disorder (GAD) based on DSM-IV criteria [14] followed by a series of newly developed items designed to measure the personal and perceived stigma associated with GAD (see Additional file 1). It also included scales or items to measure GAD social distance, exposure to people with anxiety disorders, self reported history, and past help seeking behaviours and intentions for anxiety disorders. The final section (E) presented a labelled vignette depicting a character with depression based on DSM-IV criteria [14] followed by a depression stigma measure comprising personal and perceived subscales, self reported history and past help seeking for depression and help seeking intentions. The help seeking data is the subject of a separate paper exploring help seeking for GAD and is not reported here. Details of the other measures are summarised below.

### Generalised anxiety stigma items

#### Identification of items

The methodology for identifying potential items for the Generalised Anxiety Stigma Scale (GASS) was based on that used in developing the DSS. Separate Google and Google Groups searches were undertaken using the phrases 'anxiety stigma', 'anxiety prejudice', and 'anxiety discrimination'. All results up to a maximum of 1000 for each of the six searches were recorded. From these web pages, one author extracted text relevant to stigma associated with generalised or non-specific anxiety conditions (AP) from which two authors (AP and KG) constructed potential items for the stigma scale. The resulting 205 items were then grouped into themes (AP) and discussed by a team of six mental health researchers (KG, LB, AP, JG, RR, KK). Themes which were endorsed on more than two websites were considered for inclusion in the GASS questionnaire. Since the aim was to produce a short questionnaire, overlapping themes were excluded as were themes related to stigma associated with help seeking and treatment, or themes which were by consensus of the six-person team not considered central to the concept of stigma.

The identified themes were that anxiety was *not a real medical illness*, that it was sign of *weakness*, *laziness, instability *and *self-absorption*, that people *could snap out of *anxiety if they wished, that the condition was the *fault of the person*, that those with anxiety would be *poor employees *and *a danger to others*, and that the condition was *shameful/embarrassing*.

#### The survey items

Two parallel sets of items were constructed from the ten themes identified in the item selection phase and presented together with a labelled vignette depicting a person with a Generalised Anxiety Disorder based on DSM-IV criteria (see Table 1). The first set of 10 items was intended to evaluate the respondent's personal attitudes to anxiety (GASS-Personal items); the second set of 10 items aimed to measure the respondent's perception of the attitudes of others (GASS-Perceived items). Respondents were asked to indicate to what extent they personally agreed or thought most other people would agree with each statement using the response options of Strongly Agree, Agree, Neither Agree nor Disagree, Disagree, and Strongly Disagree. The Flesch-Kincaid Reading Level for the instrument is 6.2, indicating that the scale is suitable for participants with an elementary school education or higher.

**Table 1:** Characteristics of the sample (N = 617)

*Demographic status*	N	Mean	sd
Age	558	46.60	13.25
Years of education	613	13.95	2.42
*Gender*	**N**	**%**	
Female	384	62.2%	
Male	233	37.8%	

***Clinical characteristics***	**N**	**%**	

Past history of anxiety disorder (n = 608)	111	18.3%	
	**N**	**Mean**	**sd**
Goldberg anxiety scale	617	3.01	2.73
Goldberg depression scale	617	2.23	2.28
K10 distress	609	16.29	6.96
Anxiety exposure	617	6.86	3.11

***Stigma levels***	**Mean**	**sd**	**N**

Mental illness social distance	597	5.14	3.13
Mental illness perceived stigma (DDS)	617	18.88	4.65
Depression personal stigma (DSS)	606	10.00	5.26
Depression perceived stigma (DSS)	608	20.34	5.87
Anxiety social distance scale	610	4.43	2.95
Anxiety personal stigma (GASS)	610	9.03	5.60
Anxiety perceived stigma (GASS)	608	21.51	6.61

DDS: Devaluation Discrimination Scale; GASS: Generalised Anxiety Stigma Scale; DSS: Depression Stigma Scale

### Other measures and their hypothesised relationships with the GASS

Ideally, a new measure should be validated against an existing validated measure of the construct. In the absence of the latter we used stigma scales for other types of mental disorder to evaluate the new anxiety stigma measure. Similarly, since contact with mental illness has been the most consistently demonstrated predictor of the stigma associated mental illness, we assessed the association between the new anxiety stigma measure and level of exposure to GAD. These measures are described below.

#### Social Distance Scale

This scale, which has a number of variants, is one of the most commonly employed measures of stigma and has satisfactory internal reliability and evidence of construct validity [15]. It has been used as a proxy measure of discrimination towards people with 'mental illness' [eg., [16]], 'mental health problems' [17] schizophrenia [eg., [18-20]] and depression [eg., [20]]. The current study employed the 5-item, 4 point Likert scale version of the scale [20] to evaluate the extent to which the respondent would be prepared to move next door to, socialise with, make friends with, work closely with or have a person with a mental disorder marry into the family (total score range 5 to 20). Parallel scales were employed for 'mental illness' and GAD. A higher score on this scale represents a greater level of desired personal distance. The internal reliabilities of the anxiety and general mental illness versions of the social distance scale in the current study were 0.91 and 0.89 respectively.

It was anticipated that there would be a significant association between the new GASS-Personal stigma score and the anxiety form of the Social Distance Scale. Previous research has shown a moderate positive association between the stigma associated with different disorders [21]. It was therefore hypothesised that there would be a moderate positive association between the GASS Personal stigma score and Social Distance for generic 'mental illness'. Since the concept of social distance measure is more closely related to personal than perceived stigma, it was anticipated that there would be a low correlation between the new GASS-Perceived score and all three measures of social distance.

#### Depression Stigma Scale (DSS)

This 18-item measure comprises a personal subscale (9 items) and a perceived subscale (9 items) [11,12]. Respondents were asked to indicate to what extent they personally agreed (DSS-Personal subscale) or thought most other people would agree with (DSS-Perceived subscale) each statement using a 5-point Likert scale from 'Strongly Disagree' (0) to 'Strongly Agree' (4). Subscale scores ranged from 0 to 36 with higher scores indicating greater stigma. There is good evidence for the reliability and validity of this scale [11,12,22]. The internal reliabilities of the Personal and Perceived subscales of the DSS in the current study were 0.80 and 0.86 respectively.

It was hypothesised that there would be a significant association between GASS-Personal and DSS-Personal subscales and between GASS-Perceived and DSS-Perceived subscales but low correlations between the personal and perceived subscales.

#### Devaluation-Discrimination Scale

This 12-item scale assesses perceived stigma associated with mental illness by asking respondents to indicate on a 4-point Likert-scale from 'Strongly Agree' to 'Strongly Disagree' what they believe 'most people' would think about people with a mental illness (range 0 to 36) [23-25]. Higher scores indicate greater stigma. Internal consistency has been reported previously to be 0.78 [23] and was 0.84 in the current study.

It was hypothesised that there would be a significant association between GASS-Perceived Stigma and the Devaluation Discrimination Score but a low correlation between this measure and the GASS-Personal score.

#### Level of Contact Report

Previous exposure to anxiety disorders was measured using a modified version of the Level of Contact Report [26]. In the version employed in the current study, participants were asked to endorse which of a series of 10 items listed in order of increasing exposure, best depicted their greatest level of exposure to an anxiety disorder. Items ranged from no contact (0) to personal experience of an anxiety disorder (9).

Intervention research has demonstrated that contact with people with mental illness is associated with a reduction in stigmatising attitudes [27]. There is also substantial cross-sectional evidence of an inverse association between level of contact with mental illness and stigma [28]. We therefore hypothesised that there would be a negative correlation between level of exposure to people with anxiety disorders and stigmatising attitudes (personal stigma) to anxiety disorder.

#### Past history of anxiety disorder

Self reported history of anxiety disorder was assessed using a single yes/no item: 'Have you been diagnosed with an anxiety disorder at any time in your life?' Our previous research involving community-based samples has demonstrated a significant inverse association between a previous history of depression and personal stigma [12]. Conversely, this group showed higher levels of perceived depression stigma than other members of the community [12]. We therefore expected that there would be a positive correlation between the Personal GASS scores and a past diagnosis of GAD and no or a negative correlation between Perceived GASS scores and anxiety diagnosis.

### Demographic and clinical characteristics

Self-reported gender, age, and years of education were recorded. Current anxiety and depressive symptoms were measured using the 9-item Goldberg Anxiety and 9-item Goldberg Depression Scales (range 0 to 9 for each scale) [29]. Psychological distress was measured using the 10-item Kessler Psychological Distress Scale (K10) self report scale [30]. Higher scores represent higher level of symptoms or distress.

### Analyses

The internal consistency of the anxiety stigma items was calculated using the Cronbach alpha coefficient and the factor structure by means of a Principal Components Analysis. Test-retest reliability and construct validity with other measures of stigma and contact with Generalised Anxiety Disorder were computed using Pearson's correlation coefficient, or in the case of self-reported Generalised Anxiety Disorder, a Student's t-test. The characteristics of the subset of participants undergoing retesting were compared with the remainder of the participants using the Student t-test and chi-square analyses.

## Results

### Participants

The characteristics of the total sample are displayed in Table 1. Respondents ranged in age from 18 to 68 years, over 60% were women, 27.4% had completed a Bachelor or higher degree and a substantial minority self-reported a history of anxiety disorder. The profile of the subsample on which the test-retest reliability measure was computed was not significantly different from the remainder of the total sample with respect to gender distribution (*p *= 0.87), age (*p *= .10), anxiety exposure (*p *= 0.37), psychological distress (K10) (*p *= 0.95), or anxiety symptoms (*p *= 0.56), depressive symptoms (*p *= 0.83) or self-reported history of anxiety disorder (*p *= 0.24). However, this group was significantly better educated than those who were not followed up for retest (difference = 1.02 years, t(612) = 5.09, *p *< 0.001). In addition, they showed significantly less personal anxiety stigma (GASS-Personal difference = 1.34, t(608) = -2.84, *p *= 0.005) and significantly more perceived anxiety stigma (GASS-Perceived difference = 1.38; t(617) = -2.46, *p = *0.014)

### Item responses

The mean responses and percentages of participants agreeing or strongly agreeing with each item on the GASS are shown in Table 2. Scores for items range from 0 ('Strongly Disagree') to 4 ('Strongly Agree').

**Table 2:** Response characteristics and factor loadings for the Generalised Anxiety Stigma items

	N	Mean	SD	Agree/ Strongly Agree	α (without item)	Personal factor loading	Perceived factor loading
**Personal stigma items**	**610**	**9.03**	**5.60**		**0.856**		

1. An anxiety disorder is not a real medical illness.	614	1.03	1.05	13.0%	(0.854)	0.576	

2. An anxiety disorder is a sign of personal weakness	615	0.84	0.85	6.0%	(0.836)	0.721	

3. People with an anxiety disorder could snap out of it if they wanted to.	615	0.90	0.85	6.0%	(0.836)	0.728	

4. People with an anxiety disorder should be ashamed of themselves.	613	0.44	0.65	1.5%	(0.843)	0.689	

5. People with an anxiety disorder do not make suitable employees.	613	1.11	0.90	7.8%	(0.841)	0.660	

6. People with an anxiety disorder are unstable.	612	1.41	0.99	16.7%	(0.847)	0.600	

7. People with an anxiety disorder are to blame for their problem.	612	0.78	0.78	2.9%	(0.838)	0.794	

8. People with an anxiety disorder are just lazy.	613	0.56	0.63	0.7%	(0.837)	0.771	

9. People with an anxiety disorder are a danger to others.	613	1.01	0.82	3.8%	(0.850)	0.561	

10. People with an anxiety disorder are self-centred.	613	0.96	0.88	6.4%	(0.840)	0.677	

**Perceived stigma items**	**609**	**21.51**	**6.61**		**0.909**		

1. Most people think that an anxiety disorder is not a real medical illness.	611	2.40	0.89	56.0%	(0.904)		0.673

2. Most people think that an anxiety disorder is a sign of personal weakness.	611	2.33	0.92	52.7%	(0.895)		0.803

3. Most people think that people with an anxiety disorder could snap out of it if they wanted to.	611	2.37	0.94	55.3%	(0.896)		0.796

4. Most people think that people with an anxiety disorder should be ashamed of themselves.	611	1.74	0.88	20.1%	(0.895)		0.801

5. Most people think that people with an anxiety disorder do not make suitable employees.	611	2.33	0.85	48.4%	(0.903)		0.688

6. Most people think that people with an anxiety disorder are unstable.	610	2.46	0.85	58.9%	(0.901)		0.715

7. Most people think that people with an anxiety disorder are to blame for their problem.	611	2.15	0.89	41.6%	(0.896)		0.794

8. Most people think that people with an anxiety disorder are just lazy.	611	1.80	0.92	24.5%	(0.897)		0.779

9. Most people think that people with an anxiety disorder are a danger to others.	610	1.93	0.89	28.7%	(0.906)		0.639

10. Most people think that people with an anxiety disorder are self-centred.	611	2.01	0.89	32.7%	(0.902)		0.708

The level of personal stigma was low with only a small minority of respondents endorsing each item. Only two of the 10 personal stigma items were endorsed by more than 10% of the sample. These were that Generalised Anxiety Disorder is not a real illness (13.0%) and that people with a Generalised Anxiety Disorder are unstable (16.7%). Fewer than 5% of respondents thought that people with anxiety disorder were just lazy, should be ashamed of themselves, were to blame for their problems or were a danger to others.

By contrast a substantial percentage of the sample believed that most other people would hold stigmatising attitudes to people with an anxiety disorder. Over half of the respondents endorsed the view that most other people did not believe anxiety disorder was a real medical illness, believed that they could snap out of it if they wanted to, and thought that it was a sign of personal weakness and associated with instability. All perceived stigma statements were endorsed by at least 20% of participants, with the lowest level of perceived stigma associated with shame, laziness and danger to others.

The distributions of responses to each of the items in the GASS are shown in Figure 1 of Additional File 2. There were low rates of missingness on each item, with between 3 and 8 respondents (0.5-1.3%) not providing a response to an item. Participants with missing data on one or more items were excluded from subsequent analyses.

### Factor structure

Bartlett's Test of Sphericity (χ^2 ^= 5910.8, *p *< 0.001) and a Kaiser-Meyer-Olkin value of 0.88 confirmed the suitability of investigating the factor structure of the data. A Principal Components Analysis with Varimax rotation and Kaiser normalisation combined with the Scree Plot test yielded two generalised anxiety stigma components corresponding to Personal Stigma and Perceived Stigma (see Table 1). Together, the two components accounted for 50.5% of the total variance (27.9% Perceived, 22.6% Personal). Factor loadings for the Personal Stigma Scale ranged from 0.65 to 0.80 and for the Perceived Stigma Scale from 0.57 to 0.77 with no cross loadings above 0.13. The Cronbach alphas for the 10 item Personal and 10 item Perceived GASS stigma scales were 0.86 and 0.91 respectively. No floor or ceiling effects were observed within the subscales.

### Test-retest reliability

The mean follow-up period was 17.3 (sd = 2.71) weeks. The test-retest reliability over this 4-month period was 0.58 and 0.55 for the Personal and Perceived GASS subscales respectively (*p *< 0.0001). There was no significant difference in baseline and follow-up stigma scores for either of the subscales (GASS-Personal: difference = -0.08, *t_209 _*= -0.25, *p *= 0.80; GASS-Perceived: difference = 0.39, *t_208 _*= 0.94, *p *= 0.34).

### Construct validity

#### Associations between the GASS subscales and other stigma measures

Table 3 shows the relationships between the GASS subscales and other measures of stigma. As predicted, there were significant correlations of moderate strength between The GASS-Personal subscale and existing measures of personal stigma including the DSS (*p *< .0001) and the anxiety and mental illness versions of the Social Distance scale (*p *< 0.0001) (see Table 3). Similarly, there were significant moderate correlations between the GASS-Perceived subscale and the DSS-Perceived subscale (*p *< 0.0001) and the Devaluation Discrimination Scale (*p *= 0.019). This provided evidence of convergent validity.

**Table 3:** Correlation matrix showing relationship between anxiety stigma and other measures of stigma and mental health

	1	2	3	4	5	6	7	8	9	10
1. Anxiety stigma personal (GASS)	1.00									
2. Anxiety stigma perceived (GASS)	*-0.03*	1.00								
3. Anxiety social distance scale	**0.47**	*-0.06*	1.00							
4. Depression stigma personal (DSS)	**0.66**	*-0.06*	**0.49**	1.00						
5. Depression stigma perceived (DSS)	*-0.03*	**0.67**	*0.00*	0.14	1.00					
6. Mental illness social distance	**0.39**	-0.10	**0.68**	**0.47**	*-0.03*	1.00				
7. Mental illness perceived stigma (DDS)	*0.07*	**0.42**	0.10	0.09	**0.37**	0.12	1.00			
8. Goldberg anxiety	*-0.02*	0.15	*-0.01*	*0.03*	0.12	*-0.01*	0.12	1.00		
9. Goldberg depression	*-0.02*	0.15	*-0.02*	*0.05*	0.15	*0.00*	*0.06*	**0.73**	1.00	
10. K10 distress	*-0.02*	0.19	*-0.02*	*0.03*	0.19	*0.00*	0.08	**0.69**	**0.77**	1.00
11. Anxiety exposure	**-0.30**	0.20	-0.25	-0.22	0.18	-0.19	0.08	0.23	0.21	0.28

Note: **Bold **figures correspond to absolute *r *> 0.3; *italic *figures indicate *p *> 0.05

As anticipated, there was not a significant association between the GASS-Perceived and the GASS-Personal scores (*p *= 0.40). Nor were there significant correlations between the GASS-Perceived scores and personal stigma as measured by the anxiety Social Distance Scale (*p *= 0.13) or the DSS-Personal Scale (*p *= 0.16). There was a small inverse association between the mental illness social distance and GASS-Perceived scores, but the effect was very small (*r *= -0.10, *p *= 0.02). Finally, the GASS-Personal score did not correlate significantly with the Devaluation Discrimination Scale (*r *= 0.07, *p *= 0.09). The findings provided evidence of divergent validity.

#### Associations between the GASS and level of contact

As hypothesised, there was an inverse correlation between level of contact with GAD and GASS-Personal stigma (*p *< 0.0001). Conversely, there was a small positive correlation between exposure and perceived stigma (*p *< 0.0001). Further, participants with a past history of GAD showed a lower level of personal anxiety stigma [mean difference = -3.17, 95% Confidence Interval (CI) = -4.30 to -2.04] but a higher level of perceived anxiety stigma as measured by the GASS subscales (mean difference = 1.61, 95% CI = 0.25 to 2.97).

## Discussion

The current paper describes the development and validation of the first instrument for measuring the level of the public's personal and perceived stigma for Generalised Anxiety Disorder. The resulting GASS-Personal and Perceived subscales were shown to have adequate internal consistency, 4-month test-retest reliability and construct validity.

Convergent validity was demonstrated by moderate or high correlations between: (1) the GASS-Personal scale and other measures designed to assess personal stigma or proxy discrimination including the DSS-Personal and the Social Distance Scales; (2) the GASS-Perceived stigma scale and other measures designed to assess perceived stigma including the DSS-Perceived subscale and the Devaluation-Discrimination Scale; and (3) the GASS-Personal subscale and level of contact and past history of GAD. Divergent validity was demonstrated by zero or very small correlations between: (1) the GASS-Personal measure and measures of perceived stigma including the GASS-Perceived and the Devaluation-Discrimination Scale; (2) the GASS-Perceived measure and measures of personal stigma including Social Distance and the DSS-Personal scales; and (3) the GASS-Perceived and level of contact and past history of GAD. The above findings suggest that the GASS measure may be a suitable tool for community studies of the stigma associated with Generalised Anxiety Disorder including studies of its prevalence, predictors and the interventions for reducing it.

The stability of each subscale of the GASS was demonstrated by moderately high levels of test-retest reliability and stable scores over 4 months. Evidence of such reliability is lacking for many measures of stigma or in cases where it has been measured it has been assessed over shorter periods. For example, Corrigan and his colleagues measured test-retest reliability of the Psychiatric Disability Attributions Questionnaire (PDAQ) over one day [31] and King and his collaborators measured reliability over a period of 2 weeks [32].

The percentage of participants reporting that they personally agreed with negative statements about people with GAD was substantially lower than the percentage who believed that most other people in the community would endorse stigmatising attitudes to GAD. In this respect the findings strongly resemble those previously reported by Griffiths and her collaborators for depression [12,33,34].

The relatively low level of personal stigma reported by respondents for most items is encouraging although the extent to which these findings were influenced by social desirability biases and the low response rate is unclear (see Limitations below). It is of interest that on average a greater percentage of people exhibited discriminatory responses to GAD on the Social Distance scale than endorsed stigmatising statements on the GASS. Thus 14.4% of respondents were definitely or probably unwilling to socialise with a person with GAD, and 14.4% were unwilling to make friends, 23.2% to move next door, 23.7% to work closely and 36.1% to have someone with GAD marry into the family.

It is unclear why there is a disparity in the prevalence of respondents endorsing negative views on the GASS-Personal subscale items and the GAD Social distance items. It is typically hypothesised that stigmatising attitudes underpin discriminatory behaviour [eg., [35]]. Why then are the greatest levels of proxy discriminatory responses (unwillingness to have a person with GAD marry into the family 36%) over double that of the most highly endorsed anxiety stigma item (unstable - 16.7%)? There are several possible explanations for the observed pattern of findings. One is that the items employed in the Personal subscale of the GASS do not tap the most important elements of stigma associated with GAD. The items were derived from a qualitative analysis of the text on websites identified using a public search engine. Most of this text was written by mental health stakeholders rather than by members of the public who held negative views about mental disorder. Thus, the identified sites may have more strongly represented the domain of perceived stigma than personal stigma. A second possibility is that social distance and personal stigma are underpinned by different factors. Third, perhaps some people hold non-stigmatising attitudes about individual facets of GAD (e.g., that people with GAD are not to blame, not lazy and cannot simply snap out of it) but hold the view that active interaction with a person with GAD will generate a type or level of burden (e.g., a need for emotional support, proxy stigma) that they would prefer to avoid. A final possibility is that social distance measures are more resistant to social desirability bias.

### Limitations

The primary limitation of the current study is the low response rate to the questionnaire. Although the surveys were sent to a randomly selected sample of members of the public the respondents were not a representative sample of the community. Notably the sample comprised more women than men and the respondents showed somewhat greater levels of distress than has previously been documented in the Australian population [30]. In addition, the participants in the retest subsample had significantly lower levels of personal anxiety stigma and a higher level of perceived anxiety stigma and education than those who were not included in this substudy (*p *< 0.001). The lack of representativeness of the overall sample precludes the use of the current statistics as an indicator of the prevalence of stigma associated with Generalised Anxiety Disorder. However, it does not detract from the demonstration that the GASS has adequate psychometric properties. If anything, the greater homogeneity of the sample employed for documenting the test-retest reliability of the GASS may have led to an underestimate of the true reliability. Additional research is required to further investigate the psychometric properties of the GASS including a confirmatory factor analysis of data collected from a new sample, further investigation of the scale in different populations and a demonstration of the sensitivity of the scales to change.

Another potential limitation is that self-report measures of personal stigma may underestimate the level of an individual's personal stigma and that the disparity in GASS personal and perceived stigma is a reflection of social desirability bias rather than two stigma factors. Link and his collaborators [15] have postulated that such bias may operate in self-reports of attitudes to mental illness. This possibility cannot be excluded for the GASS or any other stigma scale based on self-report. However, we can be confident that when used in a representative sample the GASS will provide a reliable indication of the minimum prevalence of anxiety stigma in the community. Secondly, previous research has demonstrated a significant association between attitudinal and physical proximity measures of stigma [36]. Further research using behavioural, physiological, or implicit measures of anxiety stigma may shed further light on the validity of the GASS measure.

## Conclusion

The GASS scale is a promising measure for use in future studies of the prevalence, nature and interventions for the stigma associated with Generalised Anxiety Disorder.

## Competing interests

The authors declare that they have no competing interests.

## Authors' contributions

KG conceived and co-designed the study, undertook statistical analyses of the data and drafted the article. PB and LB co-designed the study and critically edited the article; PB also managed the survey and undertook statistical analyses. AP co-designed the study, extracted potential items and with KG rated themes for inclusion in the GASS. AP also edited the paper. All authors approved the final version of the paper.

## Authors' information

KATHLEEN GRIFFITHS, PhD, Deputy Director, Centre for Mental Health Research, The Australian National University, Acton, Canberra, ACT, Australia, 0200. PHILIP BATTERHAM, MPH, Centre for Mental Health Research, The Australian National University, Acton, Canberra, ACT, Australia, 0200. LISA BARNEY, PhD, Centre for Mental Health Research, The Australian National University, Acton, Canberra, ACT, Australia, 0200. ALISON PARSON, BA(Hons), Centre for Mental Health Research, The Australian National University, Acton, Canberra, ACT, Australia, 0200.

## Pre-publication history

The pre-publication history for this paper can be accessed here:

http://www.biomedcentral.com/1471-244X/11/184/prepub

## Supplementary Material

Additional file 1 **Generalised Anxiety Disorder Scale**. The GASS Anxiety Disorder Scale. Click here for file

Additional file 2 **Distribution of responses to the 20 items of the GASS**. Distribution of responses to the 20 items of the GASS. Click here for file
