# Supplementary material for: The generalised anxiety stigma scale (GASS): psychometric properties in a community sample
Source: BMC Psychiatry. 2011 Nov 22;11:184. doi: 10.1186/1471-244X-11-184 (PMC3248354; doi:10.1186/1471-244X-11-184)
Supplement: Additional file 1 — Generalised Anxiety Disorder Scale. The GASS Anxiety Disorder Scale. [file 1471-244X-11-184-S1.DOCX]

# Additional file 1: Generalised Anxiety Stigma Scale (GASS)

**Now, we would like you to please read through the following description.**

*Most days over the last six months, Nicole has felt very anxious and worried about a lot of different things in her life. She finds it difficult to control how worried she gets. Most days Nicole feels ‘on edge’ and has difficulty concentrating. She is irritable and her muscles feel tense. Although she feels tired all of the time, she has difficulty sleeping. These feelings are so bad that Nicole has trouble doing ‘normal’ things. Nicole is really upset about the way she has been feeling lately. Nicole has an anxiety disorder.*

**The following statements are about anxiety disorders. Please indicate how strongly you personally agree or disagree with each statement.**

1. An anxiety disorder is not a real medical illness.

 Strongly agree

 Agree

 Neither agree nor disagree

 Disagree

 Strongly disagree

2. An anxiety disorder is a sign of personal weakness.

 Strongly agree

 Agree

 Neither agree nor disagree

 Disagree

 Strongly disagree

3. People with an anxiety disorder could snap out of it if they wanted to.

 Strongly agree

 Agree

 Neither agree nor disagree

 Disagree

 Strongly disagree

4. People with an anxiety disorder should be ashamed of themselves.

 Strongly agree

 Agree

 Neither agree nor disagree

 Disagree

 Strongly disagree

5. People with an anxiety disorder do not make suitable employees.

 Strongly agree

 Agree

 Neither agree nor disagree

 Disagree

 Strongly disagree

6. People with an anxiety disorder are unstable.

 Strongly agree

 Agree

 Neither agree nor disagree

 Disagree

 Strongly disagree

7. People with an anxiety disorder are to blame for their problem.

 Strongly agree

 Agree

 Neither agree nor disagree

 Disagree

 Strongly disagree

8. People with an anxiety disorder are just lazy.

 Strongly agree

 Agree

 Neither agree nor disagree

 Disagree

 Strongly disagree

9. People with an anxiety disorder are a danger to others.

 Strongly agree

 Agree

 Neither agree nor disagree

 Disagree

 Strongly disagree

10. People with an anxiety disorder are self-centred.

 Strongly agree

 Agree

 Neither agree nor disagree

 Disagree

 Strongly disagree

**Now we would like you to tell us what you think most other people believe. Please indicate how strongly you agree or disagree with the following statements.**

11. Most people think that an anxiety disorder is not a real medical illness.

 Strongly agree

 Agree

 Neither agree nor disagree

 Disagree

 Strongly disagree

12. Most people think that an anxiety disorder is a sign of personal weakness.

 Strongly agree

 Agree

 Neither agree nor disagree

 Disagree

 Strongly disagree

13. Most people think that people with an anxiety disorder could snap out of it if they wanted to.

 Strongly agree

 Agree

 Neither agree nor disagree

 Disagree

 Strongly disagree

14. Most people think that people with an anxiety disorder should be ashamed of themselves.

 Strongly agree

 Agree

 Neither agree nor disagree

 Disagree

 Strongly disagree

15. Most people think that people with an anxiety disorder do not make suitable employees.

 Strongly agree

 Agree

 Neither agree nor disagree

 Disagree

 Strongly disagree

16. Most people think that people with an anxiety disorder are unstable.

 Strongly agree

 Agree

 Neither agree nor disagree

 Disagree

 Strongly disagree

17. Most people think that people with an anxiety disorder are to blame for their problem.

 Strongly agree

 Agree

 Neither agree nor disagree

 Disagree

 Strongly disagree

18. Most people think that people with an anxiety disorder are just lazy.

 Strongly agree

 Agree

 Neither agree nor disagree

 Disagree

 Strongly disagree

19. Most people think that people with an anxiety disorder are a danger to others.

 Strongly agree

 Agree

 Neither agree nor disagree

 Disagree

 Strongly disagree

20. Most people think that people with an anxiety disorder are self-centred.

 Strongly agree

 Agree

 Neither agree nor disagree

 Disagree

 Strongly disagree
