# Supplementary material for: The generalised anxiety stigma scale (GASS): psychometric properties in a community sample
Source: BMC Psychiatry. 2011 Nov 22;11:184. doi: 10.1186/1471-244X-11-184 (PMC3248354; doi:10.1186/1471-244X-11-184)
Supplement: Additional file 2 — Distribution of responses to the 20 items of the GASS. Distribution of responses to the 20 items of the GASS. [file 1471-244X-11-184-S2.DOCX]

**Figure 1** Distribution of responses to the 20 items of the GASS
